# Naloxone Prescriptions Among Commercially Insured Individuals at High Risk of Opioid Overdose

**DOI:** 10.1001/jamanetworkopen.2019.3209

**Published:** 2019-05-03

**Authors:** Sarah Follman, Vineet M. Arora, Chris Lyttle, P. Quincy Moore, Mai T. Pho

**Affiliations:** 1 University of Chicago Pritzker School of Medicine, Chicago, Illinois; 2 Section of General Internal Medicine, University of Chicago Medicine, Chicago, Illinois; 3 Center for Health and the Social Sciences, University of Chicago Medicine, Chicago, Illinois; 4 Section of Emergency Medicine, University of Chicago Medicine, Chicago, Illinois; 5 Section of Infectious Diseases and Global Health, University of Chicago Medicine, Chicago, Illinois

## Abstract

**Importance:**

As opioid-related mortality continues to increase, naloxone remains a critical intervention in preventing overdose death. Opportunities to expand access through the health care setting should be optimized.

**Objective:**

To determine the characteristics of naloxone prescribing for US patients at high risk of opioid overdose.

**Design, Setting, and Participants:**

This retrospective cohort study used Truven Health MarketScan data from October 1, 2015, through December 31, 2016, of individuals with *International Statistical Classification of Diseases and Related Health Problems, Tenth Revision* codes related to opioid use, misuse, dependence, and overdose. The cohort included 138 108 commercially insured individuals aged 15 years or older in the United States with claims related to opioid misuse or dependence, opioid-related overdose, or both.

**Exposures:**

Outpatient naloxone pharmacy claims.

**Main Outcomes and Measures:**

Demographic characteristics, clinical characteristics, health care service use, and proportion prescribed naloxone were included in multivariable logistic regression analyses to test the association of opioid risk group with naloxone claim.

**Results:**

Of 138 108 high-risk individuals (mean [SD] age, 43.4 [0.4] years; 72 435 [52.4%] men), 2135 (1.5%) were prescribed naloxone. Having prior diagnoses of both opioid misuse or dependence and overdose was associated with a greater likelihood of receiving naloxone (odds ratio [OR], 2.32; 95% CI, 1.98-2.72; *P* < .001) compared with having a prior diagnosis of opioid misuse or dependence without overdose. Having a prior diagnosis of opioid overdose alone was associated with a decreased likelihood of receiving naloxone (OR, 0.73; 95% CI, 0.57-0.94; *P* = .01) compared with having a prior diagnosis of opioid misuse or dependence without overdose. Factors associated with lower naloxone prescription included being aged 30 to 44 years (OR, 0.72; 95% CI, 0.62-0.84; *P* < .001) and being from the Midwest (OR, 0.62; 95% CI, 0.54-0.71; *P* < .001) or West (OR, 0.85; 95% CI, 0.74-0.98; *P* = .03). Opioid use disorder treatment, such as use of medication-assisted therapy (OR, 1.68; 95% CI, 1.53-1.86; *P* < .001), visiting a detoxification facility (OR, 1.51; 95% CI, 1.31-1.76; *P* < .001), or receiving other substance use disorder treatment (OR, 1.16; 95% CI, 1.04-1.30; *P* = .01), were associated with increased likelihood of receiving naloxone, as were receiving outpatient care from a pain specialist (OR, 1.57; 95% CI, 1.40-1.76; *P* < .001), psychologist (OR, 1.49; 95% CI, 1.29-1.70; *P* < .001), or surgeon (OR, 1.19; 95% CI, 1.08-1.32; *P* < .001). Overall, 98.5% (n = 135 973) of high-risk patients did not received naloxone, despite many interactions with the health care system, including 88 618 hospitalizations, 229 680 emergency department visits, 298 058 internal medicine visits, and 568 448 family practice visits.

**Conclusions and Relevance:**

Patients at high risk of opioid overdose rarely received prescriptions for naloxone despite numerous interactions with the health care system. Prescribing in emergency, inpatient, and outpatient settings represents an opportunity to improve access.

## Key Points

**Question:**

How often is naloxone prescribed in a clinical setting to US patients at high risk of opioid overdose?

**Findings:**

In this cohort study using administrative data including 138 108 individuals, only 1.5% of high-risk patients, including individuals with prior opioid overdose or opioid misuse or dependence, were prescribed naloxone.

**Meaning:**

Naloxone prescribing in the health care setting represents an opportunity to improve access to this life-saving intervention.

## Teaser

This cohort study analyzes a nationwide database of commercially insured US individuals to determine how often naloxone is prescribed to individuals at high risk of opioid overdose.

## Introduction

Nationally, drug overdose deaths increased 11.4% from 2014 to 2015. Of the more than 52 000 drug overdose deaths in 2015, more than 60% involved opioids.^1^ Naloxone, an opioid antagonist, is effective at reversing potentially fatal respiratory depression in individuals who have overdosed. Naloxone is easy to use, safe, and cost-effective.^2,3^

Naloxone training and distribution have been provided since 1996 through community-based overdose education and naloxone distribution programs, such as harm-reduction organizations. Although these programs can be highly effective in reaching at-risk populations, including peers and bystanders of people who overdose, availability of such services is not widespread.^4,5^ To expand access through health care settings, both federal and state-level efforts have provided guidance and recommendations regarding increasing naloxone awareness, education, and prescribing. Such efforts include the White House Turn the Tide campaign in 2016^6^ and the Surgeon General’s Advisory on Naloxone and Opioid Overdose.^7,8^ The Centers for Disease Control and Prevention^9^ and the Federation of State Medical Boards^10^ have established guidelines for prescribing naloxone to patients considered at high risk of dying of a future overdose, including individuals with a history of opioid use disorder or prior opioid overdose and people who have been prescribed high doses of opioids for chronic pain. Additionally, most states’ naloxone access laws allow for third-party prescribing, ie, prescribing to a third party for use on someone else at risk of overdose, thereby facilitating access to the medication through bystanders.^11^

Although health care professionals play an important role in improving access to eligible patients, naloxone prescribing may remain limited in practice. A 2017 study^11^ examining clinicians’ coprescribing of naloxone for patients taking prescribed opioids found that coprescription occurred 0.02% of the time in hospital outpatient settings and 0.05% of the time in emergency department (ED) settings. Similarly, site-specific^12^ and region-specific^13^ studies have demonstrated that most physicians have little to no experience prescribing naloxone, often citing a lack of knowledge and confidence regarding overdose risk factors, dosing, administration, and relevant state legislation.

Given the well-established clinical benefits of naloxone, the high prioritization at a national level of intervention, and the continued increase in opioid-related overdoses, improved understanding of naloxone prescribing by health care professionals can inform policies and practices that expand access. To our knowledge, no published study has specifically examined naloxone prescribing using administrative data of patients on a national scale. The aim of this study is to analyze pharmacy claims for naloxone using a large database of commercially insured individuals at high risk of overdose.

## Methods

Naloxone outpatient pharmacy claims and corresponding patient claims data were extracted from the Truven Health MarketScan Research Database (Truven Health Analytics). This national database contains deidentified inpatient, outpatient, and pharmacy claims data generated by 17 million to 53 million employer-based private health plan–covered lives per year. The deidentified data were compliant with the Health Insurance Portability and Accountability Act. The University of Chicago Institutional Review Board determined this study to be exempt from review and informed consent. This study was prepared in accordance with the Strengthening the Reporting of Observational Studies in Epidemiology (STROBE) reporting guideline for cohort studies.

The study sample included individuals at high risk of opioid overdose. These individuals were identified with the *International Statistical Classification of Diseases and Related Health Problems, Tenth Revision *(*ICD-10*) codes for opioid misuse, opioid dependence and unspecified use, adverse effects of opioids, and opioid poisoning, using classifications validated by the Healthcare Cost and Utilization Project.^14^ The cohort was derived from October 1, 2015, through December 31, 2016, the period immediately after the *ICD-9* to *ICD-10* code transition. Individuals with at least 1 claim for the codes noted above were observed through December 31, 2016, or until the time of exit from the commercial insurance plan, whichever came first, and were categorized into 3 groups: (1) patients with an opioid misuse or dependence diagnosis and without a prior opioid overdose diagnosis, (2) patients without a prior opioid misuse or dependence diagnosis and with an opioid overdose diagnosis, and (3) patients with an opioid misuse or dependence diagnosis and an opioid overdose diagnosis. A full list of *ICD-10* codes used for cohort identification and grouping can be found in eTable 1 in the Supplement. Analysis began in July 2018.

For each group, we performed descriptive statistics of demographic characteristics, including age, sex, US census region (Northeast, Midwest, South, West, or unknown), insurance plan (comprehensive, preferred provider organization, health maintenance organization, point of service, or other), and total days covered. We then compared clinical characteristics and health care service use among groups, including the presence of co-occurring mental illness or substance use disorder (mood, anxiety, posttraumatic stress disorder, alcohol use disorder, or other substance use disorder) diagnoses and whether the patient had been to a detoxification facility, initiated any medication-assisted treatment (MAT), visited an ED, been admitted to a hospital, or had seen various outpatient medical professionals. A full list of *ICD-10* codes and Truven Health MarketScan Research Database identifiers used to define these variables can be found in eTables 2, 3, and 4 in the Supplement.

We linked patients to their outpatient pharmacy claims and calculated the proportion of each group that filled a prescription for naloxone during the study. We used Red Book Online (Truven Health Analytics) to extract the national drug codes for naloxone, excluding any combination drugs used for MAT purposes, such as buprenorphine-naloxone (eTable 5 in the Supplement).

### Statistical Analysis

A χ^2^ test was used to analyze differences among naloxone pharmacy claims and clinical and health care service use characteristics by group. A multivariable logistic regression model was used to test the association of opioid risk group with naloxone claim, controlling for demographic and health care service use variables. Variable selection was performed using the Pearson correlation coefficient test for pairwise correlation to assess for collinearity of the independent variables. Variables with coefficients of 0.6 or higher were removed from the model; the final variables included sex, age group, region, relationship to primary insurance holder, insurance plan, hospital admissions, ED visits, co-occurring substance use disorder diagnoses (alcohol use disorder and nonalcohol substance use disorder), presence of a co-occurring mental health disorder diagnosis (mood, anxiety, or posttraumatic stress disorder), substance use treatment (MAT, detoxification, or other), and visits to specialists. We performed the analysis using Stata statistical software version 15 (StataCorp). *P* values were 2-tailed, and significance was set at less than .01.

## Results

Among the 33 467 106 individuals in the Truven Health MarketScan Research Database between October 1, 2015, and December 31, 2016, we identified 138 108 patients (mean [SD] age, 43.4 [0.4] years; 72 435 [52.4%] men) with *ICD-10* diagnosis codes for opioid misuse, opioid dependence, or opioid overdose. All 138 108 patients were considered naloxone eligible based on prior diagnoses of opioid misuse, dependence, or overdose, which is consistent with current Centers for Disease Control and Prevention guidelines.^9^ There were 124 721 individuals (90.3%) with an opioid misuse or dependence diagnosis and without any diagnosis of opioid overdose, 8895 individuals (6.4%) without an opioid misuse or dependence diagnosis and with a diagnosis of opioid overdose, and 4492 individuals (3.3%) with an opioid misuse or dependence diagnosis and an opioid overdose diagnosis (Table 1). Individuals with diagnoses of opioid overdose alone were more likely to be women and to have been hospitalized, seen in the ED, or seen in family practice, internal medicine, and outpatient surgical procedure settings. They were less likely to have received substance use disorder treatment and were less likely to have a diagnosed co-occurring mental health or substance use disorder compared with individuals with diagnoses of opioid use disorder or dependence.

**Table 1.** Characteristics of Individuals With Opioid Misuse or Dependence or Opioid Overdose Diagnoses From October 1, 2015, to December 31, 2016

Characteristic	No. (%)				*P* Value
	Total	With Misuse or Dependence, Without Overdose	Without Misuse or Dependence, With Overdose	With Misuse or Dependence and Overdose	
**Patient Characteristic**					
Total	138 108	124 721 (90.3)	8895 (6.4)	4492 (3.3)	
Sex					
Male	72 435 (52.4)	66 137 (53.0)	3637 (40.9)	2661 (59.2)	<.001
Female	65 673 (47.6)	58 584 (47.0)	5258 (59.1)	1831 (40.8)	
Age group, y					
15-29	34 573 (25.0)	30 509 (24.5)	1697 (19.1)	2367 (52.7)	<.001
30-44	37 647 (27.3)	35 372 (28.4)	1539 (17.3)	736 (16.4)	
45-59	42 547 (30.8)	39 213 (31.4)	2534 (28.5)	800 (17.8)	
≥60	23 341 (16.9)	19 627 (15.7)	3125 (35.1)	589 (13.1)	
Region					
Northeast	24 747 (17.9)	22 338 (17.9)	1287 (14.5)	1122 (25.0)	<.001
Midwest	24 513 (17.7)	20 939 (16.8)	2440 (27.4)	1134 (25.2)	
South	69 742 (50.5)	64 208 (51.5)	3833 (43.1)	1701 (37.9)	
West	18 754 (13.6)	16 918 (13.6)	1312 (14.7)	524 (11.7)	
Unknown	352 (0.3)	318 (0.3)	23 (0.3)	11 (0.2)	
Relationship to primary policy holder					
Self	73 097 (52.9)	66 615 (53.4)	4972 (55.9)	1510 (33.6)	<.001
Spouse	39 733 (28.8)	36 145 (29.0)	2662 (29.9)	926 (20.6)	
Child or other	25 278 (18.3)	21 961 (17.6)	1261 (14.2)	2056 (45.8)	
**Health Care Characteristic**					
Insurance plan type					
Comprehensive	10 089 (7.3)	8421 (6.8)	1257 (14.1)	411 (9.1)	<.001
Preferred provider organization	82 496 (59.7)	75 063 (60.2)	4816 (54.1)	2617 (58.3)	
Health maintenance organization	12 518 (9.1)	11 227 (9.0)	824 (9.3)	467 (10.4)	
Point of service^a^	11 666 (8.4)	10 669 (8.6)	680 (7.6)	317 (7.1)	
Other	18 362 (13.3)	16 595 (13.3)	1151 (12.9)	616 (13.7)	
Insurance coverage ≥1 y	107 063 (77.5)	96 029 (77.0)	7351 (82.6)	3683 (82.0)	<.001
Hospital admissions					
1-2	36 679 (26.6)	31 099 (24.9)	3554 (40.0)	2026 (45.1)	<.001
≥3	9179 (6.6)	7280 (5.8)	828 (9.3)	1071 (23.8)	
Emergency department visits					
1-2	43 433 (31.4)	36 952 (29.6)	4527 (50.9)	1954 (43.5)	<.001
≥3	26 402 (19.1)	20 862 (16.7)	3194 (35.9)	2346 (52.2)	
Co-occurring substance use disorder					
Alcohol	20 851 (15.1)	18 802 (15.1)	592 (6.7)	1452 (32.3)	<.001
Nonalcohol	37 593 (27.2)	34 128 (27.4)	808 (9.1)	2657 (59.1)	
Co-occurring mental health disorder					
Mood	55 067 (39.9)	49 435 (39.6)	2973 (33.4)	2659 (59.2)	<.001
Anxiety	54 664 (39.6)	49 171 (39.4)	3015 (33.9)	2478 (55.2)	<.001
Posttraumatic stress	6041 (4.4)	5422 (4.3)	255 (2.9)	364 (8.1)	<.001
Substance use disorder treatment					
Medication-assisted treatment	36 122 (26.2)	34 414 (27.6)	226 (2.5)	1482 (33.0)	<.001
Detoxification	16 136 (11.7)	14 622 (11.7)	78 (0.9)	1436 (32.0)	<.001
Other	87 025 (63.0)	83 079 (66.6)	586 (6.6)	3360 (74.8)	<.001
Specialist visit					
Family practice	83 612 (60.5)	74 889 (60.0)	5815 (65.4)	2908 (64.7)	<.001
Internal medicine	55 668 (40.3)	48 973 (39.3)	4443 (49.9)	2252 (50.1)	<.001
Surgeon	42 607 (30.9)	37 297 (29.9)	4004 (45.0)	1306 (29.1)	<.001
Psychiatry^b^	29 929 (21.7)	27 165 (21.8)	1106 (12.4)	1658 (36.9)	<.001
Pain medicine	22 234 (16.1)	20 892 (16.8)	865 (9.7)	477 (10.6)	<.001
Obstetrics and gynecology	20 366 (14.7)	18 191 (14.6)	1600 (18.0)	575 (12.8)	<.001
Psychology	9132 (6.6)	8120 (6.5)	463 (5.2)	549 (12.2)	<.001

^a^ Includes traditional point-of-service plans and point of service with capitation.

^b^ Includes insurance claims linked to visits to either an adult or pediatric psychiatrist.

Among 138 108 patients identified with opioid misuse or dependence and/or overdose, 135 973 patients (98.5%) did not receive naloxone. Among the total cohort, 2135 individuals (1.5%) had claims for naloxone. Of 124 721 individuals in the group with opioid misuse or dependence diagnoses and without opioid overdose diagnoses, 1853 (1.5%) received naloxone. Of 8895 individuals in the group without opioid misuse or dependence diagnoses and with opioid overdose diagnoses, 74 (0.8%) received naloxone. Of 4492 individuals in the group with opioid misuse or dependence diagnoses and with opioid overdose diagnoses, 208 (4.6%) received naloxone. Differences among these groups were statistically significant (Figure). The patient cohort had opportunities to receive naloxone based on numerous interactions with the health care system. In the 15-month period of our analysis, the patient cohort had 88 618 hospitalizations, 229 680 ED visits, 298 058 interactions with internal medicine professionals, and 568 448 visits to family practice professionals. Notably, 69 835 patients (50.6%) had 1 or more ED visit, and 45 858 patients (33.2%) had at least 1 hospital admission. Additionally, 36 122 patients (26.2%) had received MAT, 16 136 patients (11.7%) had been to a detoxification facility, and 87 025 patients (63.1%) had received some other substance use disorder treatment.

**Figure.**
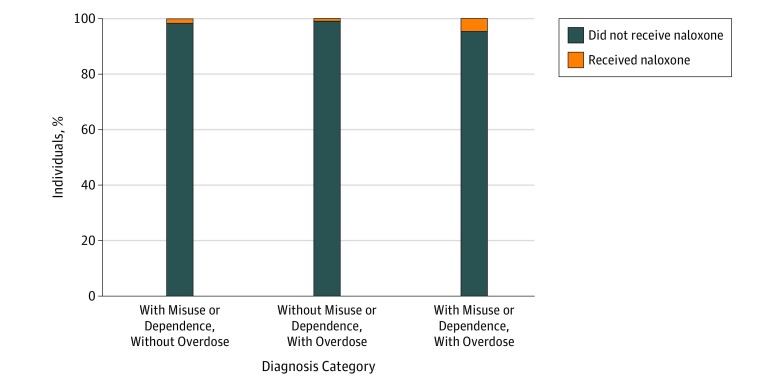
Naloxone Pharmacy Claims by Diagnosis Category

In multivariable logistic regression (Table 2), having a diagnosis of opioid misuse or dependence and a diagnosis of opioid overdose was associated with a greater likelihood of receiving naloxone (odds ratio [OR], 2.32; 95% CI, 1.98-2.72; *P* < .001) compared with the group with opioid misuse or dependence diagnoses and without opioid overdose diagnoses. In contrast, not having an opioid misuse or dependence diagnosis and having an opioid overdose diagnosis was associated with a decreased likelihood of a naloxone pharmacy claim (OR, 0.73; 95% CI, 0.57-0.94; *P* = .01).

**Table 2.** Factors Associated With Receiving Naloxone Based on a Multivariable Logistic Regression (N = 138 108)

Characteristic	Odds Ratio (95% CI)	*P* Value
With opioid misuse or dependence, without overdose (n = 124, 721)	1 [Reference]	NA
Without opioid misuse or dependence, with overdose (n = 8895)	0.73 (0.57-0.94)	.01
With opioid misuse or dependence, with overdose (n = 4492)	2.32 (1.98-2.72)	<.001
**Patient Characteristic**		
Sex		
Female	1 [Reference]	NA
Male	1.03 (0.94-1.14)	.50
Age group, y		
15-29	1.02 (0.87-1.20)	.79
30-44	0.72 (0.62-0 .84)	<.001
45-59	0.99 (0.86-1.14)	.89
≥60	1 [Reference]	NA
Region		
South	1 [Reference]	NA
Northeast	1.15 (1.03-1.29)	.01
Midwest	0.62 (0.54-0.71)	<.001
West	0.85 (0.74-0.98)	.03
Unknown	3.34 (2.06-5.43)	<.001
Relationship to primary policy holder		
Self	1 [Reference]	NA
Spouse	1.44 (1.30-1.59)	<.001
**Health Care Service Use and Clinical Characteristic**		
Health plan type		
Preferred provider organization	1 [Reference]	NA
Comprehensive	0.24 (0.13-0.42)	<.001
Health maintenance organization	0.84 (0.76-0.94)	.001
Point of service^a^	0.62 (0.51-0.76)	<.001
Other^b^	1.00 (0.85-1.18)	.98
Co-occurring substance use disorder		
None reported	1 [Reference]	NA
Alcohol	0.89 (0.78-1.01)	.07
Nonalcohol and other	1.07 (0.95-1.20)	.27
Co-occurring mental health disorder		
None reported	1 [Reference]	NA
Mood	1.10 (1.00-1.22)	.06
Anxiety	1.24 (1.12-1.36)	<.001
Posttraumatic stress	1.16 (0.98-1.39)	.09
No. of hospital admissions		
0	1 [Reference]	NA
1-2	1.10 (0.98-1.24)	.12
≥3	1.57 (1.31 1.87)	<.001
No. of emergency department visits		
0	1 [Reference]	NA
1-2	1.13 (1.01-1.25)	.03
≥3	0.95 (0.83-1.09)	.47
Substance use disorder treatment		
None reported	1 [Reference]	NA
Medication-assisted treatment	1.68 (1.53-1.86)	<.001
Detoxification facility	1.51 (1.31-1.76)	<.001
Other	1.16 (1.04-1.30)	.009
Specialist visit		
None reported	1 [Reference]	NA
Family practice	1.05 (0.95-1.15)	.34
Internal medicine	1.11 (1.01-1.22)	.03
Surgeon	1.19 (1.08-1.32)	<.001
Psychiatry	0.98 (0.88-1.09)	.70
Pain medicine	1.57 (1.40-1.76)	<.001
Obstetrics and gynecology	0.99 (0.86-1.12)	.83
Psychology	1.49 (1.29-1.70)	<.001

Abbreviation: NA, not applicable.

^a^ Includes traditional point-of-service plans and point of service with capitation.

^b^ Includes high-deductible plans and exclusive provider organizations.

Compared with older individuals (aged 45-59 years and ≥60 years), individuals aged 30 to 44 years were less likely to receive naloxone (OR, 0.72; 95% CI, 0.62-0.84; *P* < .001). Compared with people living in the South, individuals from the Northeast were more likely to receive naloxone (OR, 1.15; 95% CI, 1.03-1.29; *P* = .01), while people from the Midwest (OR, 0.62; 95% CI, 0.54-0.71; *P* < .001) and the West (OR, 0.85; 95% CI, 0.74-0.98; *P* = .03) were less likely to receive naloxone. Spouses were more likely to receive naloxone compared with primary insurance holders (OR, 1.44; 95% CI, 1.30-1.59; *P* < .001).

Compared with people in preferred provider organization plans, individuals in comprehensive insurance plans (OR, 0.24; 95% CI, 0.14-0.42; *P* < .001), health maintenance organization plans (OR, 0.84; 95% CI, 0.76-0.94; *P* = .001), and point-of-service plans (OR, 0.62; 95% CI, 0.51-0.75; *P* < .001) were less likely to receive naloxone. Compared with no diagnosed anxiety disorder, having an anxiety disorder diagnosis was associated with a greater likelihood of receiving naloxone (OR, 1.24; 95% CI, 1.12-1.36; *P* < .001). Compared with no previous substance use disorder treatment, using MAT (OR, 1.68; 95% CI, 1.53-1.86; *P* < .001), visiting a detoxification facility (OR, 1.51; 95% CI, 1.31-1.76; *P* < .001), or using other substance use disorder treatment (OR, 1.16; 95% CI, 1.04-1.30; *P* = .01) were associated with a greater likelihood of receiving naloxone. Patients with 3 or more hospital admissions (OR, 1.57; 95% CI, 1.31-1.87; *P* < .001) or 1 or 2 ED visits (OR, 1.13; 95% CI, 1.01-1.25; *P* = .03) were more likely to be prescribed naloxone compared with patients without hospital admissions or ED visits, respectively. Seeing an outpatient pain medicine physician (OR, 1.57; 95% CI, 1.40-1.76; *P* < .001), psychologist (OR, 1.49; 95% CI, 1.29-1.70; *P* < .001), or surgeon (OR, 1.19; 95% CI, 1.08-1.32; *P* < .001) was associated with increased likelihood of receiving naloxone, but seeing a family medicine practitioner (OR, 1.05; 95% CI, 0.95-1.15; *P* = .34) or obstetrician or gynecologist (OR, 0.99; 95% CI, 0.86-1.12; *P* = .83) was not. Our logistic regression model accounts for 5% of the variation in naloxone pharmacy claims (*R*^2^ = 0.05).

## Discussion

This is the first study to use a large national database of insurance claims to examine naloxone prescriptions among people at high risk of opioid overdose, to our knowledge. Our results indicate that 98.5% of eligible patients with opioid misuse, dependence, or prior overdose diagnoses were not prescribed naloxone despite numerous interactions with the health care system during which they could have received naloxone. Additionally, individuals with a diagnosis of overdose with no prior diagnosis of opioid misuse or dependence received naloxone significantly less often than individuals with a diagnosis of opioid misuse or dependence without an opioid overdose diagnosis. This is surprising, considering that prior overdose is the strongest predictor of subsequent overdose and overdose death.^15,16,17,18,19,20^

Our analysis suggests that health care visits are a missed opportunity to provide naloxone. Of note, 33.2% of the cohort had at least 1 hospital admission, yet receiving naloxone was only associated with having 3 or more hospital admissions. Similarly, 50.6% of the cohort had at least 1 ED visit, yet individuals with the greatest number of ED visits (≥3) were not more likely to receive naloxone. Additionally, 95.7% of the patients at highest risk in the cohort, individuals with diagnoses of opioid misuse or dependence and opioid overdose, had at least 1 ED visit. This highlights the importance of improving naloxone prescribing in ED and inpatient settings.

Outpatient visits also represent opportunities to consider naloxone prescribing. For example, 60.5% of the cohort saw a family medicine professional and 21.7% saw a psychiatrist, yet neither of these was significantly associated with receiving naloxone. However, seeing a psychologist was significantly associated with receiving naloxone. It is possible that seeing a psychologist may increase an individual’s likelihood of being referred to other specialists or to intensive outpatient or partial hospitalization substance use disorder treatment programs.

Understanding the barriers to physician prescribing within specialties may help efforts to improve prescribing rates in the outpatient setting. Some literature has described attitudes and behaviors toward naloxone among clinicians and other health care professionals. For example, a 2018 study^13^ that surveyed prescribers at select Midwest regional health centers attributed the low rates of naloxone prescribing to a lack of awareness regarding state naloxone laws and low self-confidence regarding dosing and prescribing best practices. A 2016 study^21^ showed an association of lack of confidence in risk assessment and prescribing knowledge gaps with low naloxone prescribing rates, a finding that persisted even when health care professional awareness of naloxone and willingness to prescribe were favorable. In our analysis, a substantial proportion of high-risk patients were seen by obstetric professionals. Given the increased incidence of maternal opioid use disorder and neonatal abstinence syndrome, increasing opportunities for education about opioid use disorder screening and the spectrum of treatment and services, including naloxone, should be considered for a broad array of health care professionals.^22,23^ The low rates of prescribing appear across different specialties and practice settings, and solutions to these shortfalls may vary based on the unique characteristics of each clinical context.

The observable difference in naloxone distribution by region of the United States may have meaningful implications. Recent Centers for Disease Control and Prevention statistics^24^ show that in 2017, the Northeast fared better in terms of the year-over-year change in overdose fatalities, with reduced overdose fatality rates in Northeastern states, such as Vermont, Massachusetts, and Connecticut. In contrast, fatalities in the Midwest, West, and Mid-Atlantic regions increased during this period (10%-20% increases in most areas, with increases as high as 27% in some areas). While such decreases are likely multifactorial and should not be solely attributed to naloxone prescribing, our findings suggest that policy makers in the Midwest and West could consider supporting more aggressive naloxone distribution efforts. Rural and urban areas may experience different naloxone access, and although our data set does not include this as a variable, it is important to account for the potential difference when considering policy.

We found that individuals aged 30 to 44 years were less likely to receive naloxone than older individuals. This may be a result of younger individuals seeking less health care, whereas older individuals generally have more touchpoints with health care professionals and institutions, thereby giving them more opportunities to receive a prescription for naloxone.^25^ This underscores the importance of alternative approaches to naloxone distribution for targeting younger patients who may have few, if any, additional health care interactions.

### Limitations

Our analysis has several limitations. First, claims data are subject to data coding limitations. However, our study period coincided with the onset of *ICD-10* diagnostic and billing codes, which have been shown to be more comprehensive and specific in terms of classifying opioid use and overdose.^14^ Second, while our study includes a large, nationally representative sample of patients predominantly enrolled in employer-based preferred provider organization plans, it does not include generally lower-income individuals covered by Medicaid or certain Medicare plans or uninsured individuals, and our findings may not be generalizable to these populations.

Another limitation is that substance use and mental health disorders are often undercoded in claims data because of associated stigma.^26^ More than 2.1 million people in the United States were estimated to have opioid use disorders in 2016, so we suspect that our prevalence of opioid misuse and overdose is underestimated, whereas naloxone prescribing rates are overestimated.^27^ Stigma intersects with various aspects of patient and clinician behavior and may impact the receipt of naloxone. For example, in qualitative studies^28,29^ of individuals obtaining naloxone at a pharmacy in New England states, perceived stigma from the pharmacists and fear of future consequences of requesting naloxone emerged as barriers. Another coding limitation is that initiation of substance use disorder treatment could be associated with receiving an *ICD-10* diagnosis code related to opioid misuse or dependence, which could contribute to some degree of collinearity in our regression model.

Additionally, our analysis underestimates the true distribution and use of naloxone, as many patients may receive naloxone through overdose education and naloxone distribution programs and other venues where insurance is not billed.^15,30,31^ Overdose education and naloxone distribution programs were the earliest programs to offer naloxone to at-risk individuals and their peers in the late 1990s and now include large national organizations, including the Veterans Administration, law enforcement, and hospital- and clinic-based programs. Additionally, patients could pay out of pocket for naloxone, bypassing an insurance claim, or they could receive naloxone through methadone clinics or other treatment settings that have historically had separate funding sources and billing processes.^32^ Our analysis does not aim to characterize the precise size and scope of such programs but attempts to identify additional opportunities in the health care system through which patients may gain access to naloxone.

## Conclusions

Our results suggest that most individuals at high risk of opioid overdose do not receive naloxone through direct prescribing. Clinicians can address this gap by regularly prescribing naloxone to eligible patients. To address barriers to prescribing, hospital systems and medical schools can support clinicians by improving education on screening and treating substance use disorders, clarifying legal concerns, and developing policies and protocols to guide implementation of increased prescribing. Health care systems can also create or strengthen processes to encourage naloxone prescribing. For example, the development of clinical protocols to facilitate ED prescribing and support outpatient health care professionals, such as family medicine clinicians and obstetricians, should be considered. Future policy interventions could include increasing funding in geographic regions that prescribe naloxone less frequently than other areas and incentivizing clinical programs and services that reach younger patient populations. Promoting naloxone prescribing by leveraging ongoing health care interactions with high-risk patients represents an underused and potentially effective strategy to reduce opioid overdose fatality.

## Notes

Supplement. **eTable 1.** *ICD-10* Codes for Opioid Misuse, Dependence, and Overdose **eTable 2.** MarketScan Codes Used to Identify Emergency Department Visits **eTable 3.** MarketScan Codes Used to Identify Outpatient Providers **eTable 4.** *ICD-10* Codes Used to Identify Other Substance Use Disorders and Mental Health Diagnoses **eTable 5.** Red Book Naloxone Medications

## References

[1] Seth P, Scholl L, Rudd RA, Bacon S Overdose deaths involving opioids, cocaine, and psychostimulants—United States, 2015-2016. MMWR Morb Mortal Wkly Rep. 2018;67(12):-. doi:10.15585/mmwr.mm6712a1 29596405 PMC5877356

[2] Lewis CR, Vo HT, Fishman M Intranasal naloxone and related strategies for opioid overdose intervention by nonmedical personnel: a review. Subst Abuse Rehabil. 2017;8:79-95. doi:10.2147/SAR.S101700 29066940 PMC5644601

[3] Coffin PO, Sullivan SD Cost-effectiveness of distributing naloxone to heroin users for lay overdose reversal. Ann Intern Med. 2013;158(1):1-9. doi:10.7326/0003-4819-158-1-201301010-00003 23277895

[4] Des Jarlais DC Harm reduction in the USA: the research perspective and an archive to David Purchase. Harm Reduct J. 2017;14(1):51. doi:10.1186/s12954-017-0178-6 28747189 PMC5530540

[5] Des Jarlais DC, Nugent A, Solberg A, Feelemyer J, Mermin J, Holtzman D Syringe service programs for persons who inject drugs in urban, suburban, and rural areas—United States, 2013. MMWR Morb Mortal Wkly Rep. 2015;64(48):1337-1341. doi:10.15585/mmwr.mm6448a3 26655918

[6] Murthy VH Opioid epidemic: we all have a role in turning the tide. https://obamawhitehouse.archives.gov/blog/2016/10/05/opioid-epidemic-we-all-have-role-turning-tide. Accessed March 20, 2019.

[7] US Department of Health and Human Services Surgeon General’s advisory on naloxone and opioid overdose. https://www.surgeongeneral.gov/priorities/opioid-overdose-prevention/naloxone-advisory.html. Accessed March 20, 2019.

[8] Adams JM Increasing naloxone awareness and use: the role of health care practitioners. JAMA. 2018;319(20):2073-2074. doi:10.1001/jama.2018.4867 29621389

[9] Centers for Disease Control and Prevention CDC guideline for prescribing opioids for chronic pain. https://www.cdc.gov/drugoverdose/pdf/guidelines_at-a-glance-a.pdf. Accessed March 20, 2019.

[10] Federation of State Medical Boards Guidelines for the chronic use of opioid analgesics. https://www.fsmb.org/siteassets/advocacy/policies/opioid_guidelines_as_adopted_april-2017_final.pdf. Accessed March 20, 2019.

[11] Davis C, Carr D State legal innovations to encourage naloxone dispensing. J Am Pharm Assoc (2003). 2017;57(2S):S180-S184. doi:10.1016/j.japh.2016.11.007 28073688

[12] Ebbert JO, Philpot LM, Clements CM, Attitudes, beliefs, practices, and concerns among clinicians prescribing opioids in a large academic institution. Pain Med. 2018;19(9):1790-1798. doi:10.1093/pm/pnx140 29177439

[13] Okoro ON, Bastianelli KM, Wen YF, Bilden EF, Konowalchuk BK, Schneiderhan ME Awareness of state legislation on naloxone accessibility associated with willingness to prescribe naloxone. Subst Abus. 2018;39(1):14-20. doi:10.1080/08897077.2017.1356787 28727957

[14] Moore BJ, Barrett ML Case study: exploring how opioid-related diagnosis codes translate from *ICD-9-CM* to *ICD-10-CM*. https://www.hcup-us.ahrq.gov/datainnovations/ICD-10CaseStudyonOpioid-RelatedIPStays042417.pdf. Accessed March 20, 2019.

[16] Mueller SR, Walley AY, Calcaterra SL, Glanz JM, Binswanger IA A review of opioid overdose prevention and naloxone prescribing: implications for translating community programming into clinical practice. Subst Abus. 2015;36(2):240-253. doi:10.1080/08897077.2015.1010032 25774771 PMC4470731

[17] Darke S, Mills KL, Ross J, Teesson M Rates and correlates of mortality amongst heroin users: findings from the Australian Treatment Outcome Study (ATOS), 2001-2009. Drug Alcohol Depend. 2011;115(3):190-195. doi:10.1016/j.drugalcdep.2010.10.021 21130585

[18] Coffin PO, Tracy M, Bucciarelli A, Ompad D, Vlahov D, Galea S Identifying injection drug users at risk of nonfatal overdose. Acad Emerg Med. 2007;14(7):616-623. doi:10.1197/j.aem.2007.04.005 17554010

[19] Evans JL, Tsui JI, Hahn JA, Davidson PJ, Lum PJ, Page K Mortality among young injection drug users in San Francisco: a 10-year follow-up of the UFO study. Am J Epidemiol. 2012;175(4):302-308. doi:10.1093/aje/kwr318 22227793 PMC3271816

[20] Stoové MA, Dietze PM, Jolley D Overdose deaths following previous non-fatal heroin overdose: record linkage of ambulance attendance and death registry data. Drug Alcohol Rev. 2009;28(4):347-352. doi:10.1111/j.1465-3362.2009.00057.x 19594787

[21] Wines JDJ Jr, Saitz R, Horton NJ, Lloyd-Travaglini C, Samet JH Overdose after detoxification: a prospective study. Drug Alcohol Depend. 2007;89(2-3):161-169. doi:10.1016/j.drugalcdep.2006.12.019 17280803

[22] Wilson JD, Spicyn N, Matson P, Alvanzo A, Feldman L Internal medicine resident knowledge, attitudes, and barriers to naloxone prescription in hospital and clinic settings. Subst Abus. 2016;37(3):480-487. doi:10.1080/08897077.2016.1142921 26820604 PMC6400459

[23] Haight SC, Ko JY, Tong VT, Bohm MK, Callaghan WM Opioid use disorder documented at delivery hospitalization—United States, 1999-2014. MMWR Morb Mortal Wkly Rep. 2018;67(31):845-849. doi:10.15585/mmwr.mm6731a1 30091969 PMC6089335

[24] Ko JY, Patrick SW, Tong VT, Patel R, Lind JN, Barfield WD Incidence of neonatal abstinence syndrome—28 states, 1999-2013. MMWR Morb Mortal Wkly Rep. 2016;65(31):799-802. doi:10.15585/mmwr.mm6531a2 27513154

[25] National Vital Statistics System Provisional drug overdose death counts. https://www.cdc.gov/nchs/nvss/vsrr/drug-overdose-data.htm. Accessed March 20, 2019.

[26] National Center for Health Statistics Ambulatory care use and physician office visits. https://www.cdc.gov/nchs/fastats/physician-visits.htm. Accessed March 20, 2019.

[27] Wollschlaeger BA, Willson TM, Montejano LB, Ronquest NA, Nadipelli VR Characteristics and treatment patterns of US commercially insured and Medicaid patients with opioid dependence or abuse. J Opioid Manag. 2017;13(4):207-220. doi:10.5055/jom.2017.0389 28953313

[28] Ahrnsbrak R, Bose J, Hedden SL, Lipari RN, Park-Lee E; Substance Abuse and Mental Health Services Administration Key substance use and mental health indicators in the United States: results from the 2016 National Survey on Drug Use and Health. https://www.samhsa.gov/data/sites/default/files/NSDUH-FFR1-2016/NSDUH-FFR1-2016.htm. Accessed March 20, 2019.

[29] Donovan E, Case P, Bratberg JP, Beliefs associated with pharmacy-based naloxone: a qualitative study of pharmacy-based naloxone purchasers and people at risk for opioid overdose [published online February 11, 2019]. J Urban Health. 30747371 10.1007/s11524-019-00349-1 PMC6565759

[30] Green TC, Case P, Fiske H, Perpetuating stigma or reducing risk: perspectives from naloxone consumers and pharmacists on pharmacy-based naloxone in 2 states. J Am Pharm Assoc (2003). 2017;57(2S):S19-27.e4. doi:10.1016/j.japh.2017.01.013 28214219

[31] Wheeler E, Jones TS, Gilbert MK, Davidson PJ; Centers for Disease Control and Prevention Opioid overdose prevention programs providing naloxone to laypersons—United States, 2014. MMWR Morb Mortal Wkly Rep. 2015;64(23):631-635. 26086633 PMC4584734

[32] Walley AY, Xuan Z, Hackman HH, Opioid overdose rates and implementation of overdose education and nasal naloxone distribution in Massachusetts: interrupted time series analysis. BMJ. 2013;346:f174. doi:10.1136/bmj.f174 23372174 PMC4688551

[33] Morgan L, Weaver M, Sayeed Z, Orr R The use of prescription monitoring programs to reduce opioid diversion and improve patient safety. J Pain Palliat Care Pharmacother. 2013;27(1):4-9. doi:10.3109/15360288.2012.738288 23190160

