# Supplementary material for: Naloxone Prescriptions Among Commercially Insured Individuals at High Risk of Opioid Overdose
Source: JAMA Netw Open. 2019 May 3;2(5):e193209. doi: 10.1001/jamanetworkopen.2019.3209 (PMC6503491; doi:10.1001/jamanetworkopen.2019.3209)
Supplement: Supplement. — eTable 1. ICD-10 Codes for Opioid Misuse, Dependence, and Overdose eTable 2. MarketScan Codes Used to Identify Emergency Department Visits eTable 3. MarketScan Codes Used to Identify Outpatient Providers eTable 4. ICD-10 Codes Used to Identify Other Substance Use Disorders and Mental Health Diagnoses eTable 5. Red Book Naloxone Medications [file jamanetwopen-2-e193209-s001.pdf]

Supplementary Online Content

Follman S, Arora VM, Lyttle C, Moore PQ, Pho MT. Naloxone prescriptions among commercially insured individuals at high risk of opioid overdose. *JAMA Netw Open*. 2019;2(5):e193209. doi:10.1001/jamanetworkopen.2019.3209

eTable 1. *ICD-10* Codes for Opioid Misuse, Dependence, and Overdose
eTable 2. MarketScan Codes Used to Identify Emergency Department Visits
eTable 3. MarketScan Codes Used to Identify Outpatient Providers
eTable 4. *ICD-10* Codes Used to Identify Other Substance Use Disorders and Mental Health Diagnoses
eTable 5. Red Book Naloxone Medications

This supplementary material has been provided by the authors to give readers additional information about their work.

© 2019 Follman S et al. *JAMA Network Open.*

eTable 1. ***ICD-10*** **Codes for Opioid Misuse, Dependence, and Overdose**

**Opioid Misuse & Dependence ICD-10 Codes:**	
**Opioid Misuse (Abuse):**	
F1110	Opioid abuse, uncomplicated
F11120	Opioid abuse with intoxication, uncomplicated
F11121	Opioid abuse with intoxication delirium
F11122	Opioid abuse with intoxication with perceptual disturbance
F11129	Opioid abuse with intoxication, unspecified
F1114	Opioid abuse with opioid-induced mood disorder
F11150	Opioid abuse with opioid-induced psychotic disorder with delusions
F11151	Opioid abuse with opioid-induced psychotic disorder with hallucinations
F11159	Opioid abuse with opioid-induced psychotic disorder, unspecified
F11181	Opioid abuse with opioid-induced sexual dysfunction
F11182	Opioid abuse with opioid-induced sleep disorder
F11188	Opioid abuse with other opioid-induced disorder
F1119	Opioid abuse with unspecified opioid-induced disorder
**Opioid Dependence and Unspecified Use:**	
F1120	Opioid dependence, uncomplicated
F11220	Opioid dependence with intoxication, uncomplicated
F11221	Opioid dependence with intoxication delirium
F11222	Opioid dependence with intoxication with perceptual disturbance
F11229	Opioid dependence with intoxication, unspecified
F1123	Opioid dependence with withdrawal
F1124	Opioid dependence with opioid-induced mood disorder
F11250	Opioid dependence with opioid-induced psychotic disorder with delusions
F11251	Opioid dependence with opioid-induced psychotic disorder with hallucinations
F11259	Opioid dependence with opioid-induced psychotic disorder, unspecified
F11281	Opioid dependence with opioid-induced sexual dysfunction
F11282	Opioid dependence with opioid-induced sleep disorder
F11288	Opioid dependence with other opioid-induced disorder
F1129	Opioid dependence with unspecified opioid-induced disorder
F1190	Opioid use, unspecified, uncomplicated
F11920	Opioid use, unspecified with intoxication, uncomplicated
F11921	Opioid use, unspecified with intoxication delirium
F11922	Opioid use, unspecified with intoxication with perceptual disturbance
F11929	Opioid use, unspecified with intoxication, unspecified
F1193	Opioid use, unspecified with withdrawal
F1194	Opioid use, unspecified with opioid-induced mood disorder
F11950	Opioid use, unspecified with opioid-induced psychotic disorder with delusions

© 2019 Follman S et al. *JAMA Network Open.*

F11951	Opioid use, unspecified with opioid-induced psychotic disorder with hallucinations
F11959	Opioid use, unspecified with opioid-induced psychotic disorder, unspecified
F11981	Opioid use, unspecified with opioid-induced sexual dysfunction
F11982	Opioid use, unspecified with opioid-induced sleep disorder
F11988	Opioid use, unspecified with other opioid-induced disorder
F1199	Opioid use, unspecified with unspecified opioid-induced disorder
**OVERDOSE ICD-10 Codes:**	
**Adverse effects of Opioids:**	
T400X5A	Adverse effect of opium, initial encounter
T400X5D	Adverse effect of opium, subsequent encounter
T400X5S	Adverse effect of opium, sequela
T402X5A	Adverse effect of other opioids, initial encounter
T402X5D	Adverse effect of other opioids, subsequent encounter
T402X5S	Adverse effect of other opioids, sequela
T403X5A	Adverse effect of methadone, initial encounter
T403X5D	Adverse effect of methadone, subsequent encounter
T403X5S	Adverse effect of methadone, sequela
T404X5A	Adverse effect of other synthetic narcotics, initial encounter
T404X5D	Adverse effect of other synthetic narcotics, subsequent encounter
T404X5S	Adverse effect of other synthetic narcotics, sequela
T40605A	Adverse effect of unspecified narcotics, initial encounter
T40605D	Adverse effect of unspecified narcotics, subsequent encounter
T40605S	Adverse effect of unspecified narcotics, sequela
T40695A	Adverse effect of other narcotics, initial encounter
T40695D	Adverse effect of other narcotics, subsequent encounter
T40695S	Adverse effect of other narcotics, sequela
**Opioid Poisoning:**	
T400X1A	Poisoning by opium, accidental (unintentional), initial encounter
T400X1D	Poisoning by opium, accidental (unintentional), subsequent encounter
T400X1S	Poisoning by opium, accidental (unintentional), sequela
T400X4A	Poisoning by opium, undetermined, initial encounter
T400X4D	Poisoning by opium, undetermined, subsequent encounter

© 2019 Follman S et al. *JAMA Network Open.*

T400X4S	Poisoning by opium, undetermined, sequela
T401X1 A	Poisoning by heroin, accidental (unintentional), initial encounter
T401X1 D	Poisoning by heroin, accidental (unintentional), subsequent encounter
T401X1S	Poisoning by heroin, accidental (unintentional), sequela
T401X4 A	Poisoning by heroin, undetermined, initial encounter
T401X4 D	Poisoning by heroin, undetermined, subsequent encounter
T401X4S	Poisoning by heroin, undetermined, sequela
T402X1 A	Poisoning by other opioids, accidental (unintentional), initial encounter
T402X1 D	Poisoning by other opioids, accidental (unintentional), subsequent encounter
T402X1S	Poisoning by other opioids, accidental (unintentional), sequela
T402X4 A	Poisoning by other opioids, undetermined, initial encounter
T402X4 D	Poisoning by other opioids, undetermined, subsequent encounter
T402X4S	Poisoning by other opioids, undetermined, sequela
T403X1 A	Poisoning by methadone, accidental (unintentional), initial encounter
T403X1 D	Poisoning by methadone, accidental (unintentional), subsequent encounter
T403X1S	Poisoning by methadone, accidental (unintentional), sequela
T403X4 A	Poisoning by methadone, undetermined, initial encounter
T403X4 D	Poisoning by methadone, undetermined, subsequent encounter
T403X4S	Poisoning by methadone, undetermined, sequela
T404X1 A	Poisoning by other synthetic narcotics, accidental (unintentional), initial encounter
T404X1 D	Poisoning by other synthetic narcotics, accidental (unintentional), subsequent encounter
T404X1S	Poisoning by other synthetic narcotics, accidental (unintentional), sequela
T404X4 A	Poisoning by other synthetic narcotics, undetermined, initial encounter
T404X4 D	Poisoning by other synthetic narcotics, undetermined, subsequent encounter
T404X4S	Poisoning by other synthetic narcotics, undetermined, sequela
T40601A	Poisoning by unspecified narcotics, accidental (unintentional), initial encounter
T40601D	Poisoning by unspecified narcotics, accidental (unintentional), subsequent encounter
T40601S	Poisoning by unspecified narcotics, accidental (unintentional), sequela
T40604A	Poisoning by unspecified narcotics, undetermined, initial encounter
T40604D	Poisoning by unspecified narcotics, undetermined, subsequent encounter
T40604S	Poisoning by unspecified narcotics, undetermined, sequela
T40691A	Poisoning by other narcotics, accidental (unintentional), initial encounter
T40691D	Poisoning by other narcotics, accidental (unintentional), subsequent encounter

© 2019 Follman S et al. *JAMA Network Open.*

T40691S	Poisoning by other narcotics, accidental (unintentional), sequela
T40694A	Poisoning by other narcotics, undetermined, initial encounter
T40694D	Poisoning by other narcotics, undetermined, subsequent encounter
T40694S	Poisoning by other narcotics, undetermined, sequela

© 2019 Follman S et al. *JAMA Network Open.*

eTable 2. MarketScan Codes Used to Identify Emergency Department Visits

**ED visits were defined based on 3 variable domains:**			
**1. Attachment G: PROCGRP**			
111	Emergency Department Visits		
114	ER visits, Other		
**2. Attachment H: REVCODE**			
450	Emergency Room - general classification		
451	Emergency room - emtala emergency room screening		
452	Emergency room - ER beyond emtala screening		
456	Emergency room - urgent care		
459	Emergency room - other		
981	Professional fees - emergency room		
**3. Attachment K: STDPLAC**			
23	Emergency room - Hospital		
If patients were captured by any of these codes, they were included in the ED interaction flag			

© 2019 Follman S et al. *JAMA Network Open.*

eTable 3. MarketScan Codes Used to Identify Outpatient Providers

**# visits with select outpatient providers were identified with the following codes:**			
**Attachment L - STDPROV**			
140	Pain mgmt/Pain medicine		
202	Osteopathic Medicine		
204	Internal Medicine		
240	Family Practice		
320	Obstetrics and Gynecology		
365	Psychiatry		
400	Pediatrician (general)		
458	Child Psychiatry		
860	Psychologist		
"Surgery" included any of the following:			
500	Surgeon (general)		
505	Surgical specialist		
510	Colon & Rectal Surgery		
520	Neurological Surgery		
530	Orthopedic Surgery		
535	Abdominal Surgery		
520	Cardiovascular Surgery		
550	General Vascular Surgery		
555	Head and Neck Surgery		
560	Pediatric Surgery		
565	Surgical Critical Care		
570	Transplant Surgery		
575	Traumatic Surgery		
580	Cardiothoracic Surgery		
585	Thoracic Surgery		
"Chemical Dependency / Mental Health Tx" included:			
15	Treatment Center		
20	Mental Health/Chemical Dep		
21	Mental Health Facilities		
22	Chem Depend Treatment Ctr		
23	Mental Hlth/Chem Dep Day Care		
35	Residential Treatment Center		

© 2019 Follman S et al. *JAMA Network Open.*

eTable 4: *ICD-10* Codes Used to Identify Other Substance Use Disorders and Mental Health Diagnoses

**Anxiety Disorders:**	
F40	Phobic Anxiety Disorders
F41	Other Anxiety Disorders
F42	Obsessive-compulsive disorder
F43.1	Post-traumatic Stress Disorder (PTSD)
**Mood Disorders:**	
F30	Manic Episode
F31	Bipolar Disorder
F32	Major Depressive Disorder, single episode
F33	Major depressive disorder, recurrent
F34	Persistent mood disorder
F39	Unspecified Mood disorder
**Other Substance Use Disorders:**	
**Alcohol:**	
F10	Alcohol Related Disorders
G62.1	Alcoholic polyneuropathy
I42.6	Alcoholic cardiomyopathy
K29.20	Alcoholic gastritis without bleeding
K29.21	Alcoholic gastritis with bleeding
K70.0	Alcoholic fatty liver
K70.10	Acute alcoholic hepatitis
K70.9	Chronic alcoholic hepatitis
K70.30	Alcoholic cirrhosis of liver without ascites
T51.0X1A<br>T51.0X2A<br>T51.0X3A<br>T51.0X4A	Toxic effect of ethanol
T51.1X1A<br>T51.1X2A<br>T51.1X3A<br>T51.1X4A	Toxic effect of methanol
T51.2X1A<br>T51.2X2A<br>T51.2X3A<br>T51.2X4A	Toxic effect of 2-Propanol
T51.3X1A<br>T51.3X2A<br>T51.3X3A<br>T51.3X4A	Toxic effect of fusel oil
T51.8X1A<br>T51.8X2A<br>T51.8X3A<br>T51.8X4A	Toxic effect of other specified alcohols
T51.9X1A	Toxic effect of unspecified alcohol

© 2019 Follman S et al. *JAMA Network Open.*

T51.9X2A<br>T51.9X3A<br>T51.9X4A	
**Non-alcohol:**	
F12	Cannabis Related Disorders
F13	Sedative, hypnotic, or anxiolytic related disorders
F14	Cocaine related disorders
F15	Other stimulant related disorders
F16	Hallucinogen related disorders
F18	Inhalant related disorders
F19	Other psychoactive substance related disorders

© 2019 Follman S et al. *JAMA Network Open.*

eTable 5. Red Book Naloxone Medications

The following medications were included in our analyses:

**GENNME**	**PRODNME**
NALOXONE HYDROCHLORIDE	EVZIO
NALOXONE HYDROCHLORIDE	NALOXONE HCL
NALOXONE HYDROCHLORIDE	NALOXONE HCL NOVAPLUS
NALOXONE HYDROCHLORIDE	NARCAN
NALOXONE HYDROCHLORIDE	PREMIERPRO RX NALOXONE HCL
NALOXONE HYDROCHLORIDE	NALOXONE HCL ANHYDROUS
NALOXONE HYDROCHLORIDE	NALOXONE HCL DIHYDRATE

The following medications were excluded in our analyses:

**GENNME**	**PRODNME**
BUPRENORPHINE/NALOXONE	BUNAVAIL
BUPRENORPHINE/NALOXONE	BUPRENORPHINE-NALOXONE
BUPRENORPHINE/NALOXONE	SUBOXONE
BUPRENORPHINE/NALOXONE	ZUBSOLV
NALOXONE HYDROCHLORIDE/PENTAZOCINE HYDROCHLORIDE	NALOXONE/PENTAZOCINE
NALOXONE HYDROCHLORIDE/PENTAZOCINE HYDROCHLORIDE	PENTAZOCIN/NALOXONE
NALOXONE HYDROCHLORIDE/PENTAZOCINE HYDROCHLORIDE	PENTAZOCINE HCL-NALOXONE HCL
NALOXONE HYDROCHLORIDE/PENTAZOCINE HYDROCHLORIDE	TALWIN NX
NALOXONE HYDROCHLORIDE	NARCAN NEONATAL

© 2019 Follman S et al. *JAMA Network Open.*
